# Multi-Step Procedure for Predicting Early-Age Thermal Cracking Risk in Mass Concrete Structures

**DOI:** 10.3390/ma17153700

**Published:** 2024-07-26

**Authors:** Barbara Klemczak, Aneta Smolana

**Affiliations:** Department of Structural Engineering, Silesian University of Technology, 44-100 Gliwice, Poland; aneta.smolana@polsl.pl

**Keywords:** cement hydration, temperature, thermal cracking risk, mass concrete, early age concrete, prediction methods, concrete wall

## Abstract

Early-age cracking in mass concrete structures resulting from thermal stress is a well-documented phenomenon that impacts their functionality, durability, and integrity. The primary cause of these cracks is the uneven temperature rise within the structure due to the exothermic nature of cement hydration. Assessing the likelihood of cracking involves comparing the tensile strength or strain capacity of the concrete with the stresses or strains experienced by the structure. Challenges in evaluating the risk of thermal cracking in mass concrete structures stem from various material and technological factors that influence the magnitude and progression of hydration heat-induced temperature and thermal stress. These complexities can be addressed through numerical analysis, particularly finite element analysis (FEA), which offers comprehensive modeling of early-age effects by considering all pertinent material and technological variables. However, employing FEA poses challenges such as the requirement for numerous input parameters, which may be challenging to define, and the need for specialized software not commonly available to structural engineers. Consequently, the necessity for such advanced modeling, which demands significant time investment, may not always be warranted and should be initially assessed through simpler methods. This is primarily because the definition of massive structures—those susceptible to adverse effects such as cracking due to temperature rise from hydration heat—is not precise. To address these challenges, the authors propose a three-step method for evaluating structures in this regard. The first step involves a simplified method for the classification of massive structures. The second step entails estimating hardening temperatures and levels of thermal stress using straightforward analytical techniques. The third step, reserved for structures identified as having a potential risk of early thermal cracks, involves numerical modeling. The outlined procedure is illustrated with an example application, demonstrating its practicality in analyzing a massive concrete wall constructed on the foundation.

## 1. Introduction

Cracks in early-age mass concrete structures caused by hydration heat are a well-known phenomenon that affects their serviceability, durability, and tightness [1,2,3,4,5,6,7,8,9]. Typically known examples of mass concrete structures prone to this issue include dams, nuclear power plant containments, large foundations, massive bridge piers and abutments, tunnels and tunnel linings, water-retaining structures, tanks, thick concrete slabs, and offshore structures [10,11,12,13,14,15]. Dams are most commonly associated with massive structures and were the first structures in which problems with the heat of hydration were noticed [4]. Dams, particularly gravity and arch dams, are highly susceptible to thermal cracking due to the large volumes of concrete involved [16,17,18,19]. Additionally, the curved shape of arch dams imposes additional constraints that can result in thermal cracking. Similarly, nuclear power plant containments, with their massive concrete walls and foundations designed to contain radioactive materials, face significant thermal gradients during curing, making them prone to cracking. Further, large foundations for skyscrapers, bridges, and industrial plants are also vulnerable to thermal cracking. The large pours of concrete and thick cross sections required for these foundations generate substantial internal heat. Similarly, massive bridge piers and abutments, tunnels, and tunnel linings can experience thermal stresses due to the large volumes of concrete used. Water-retaining structures, such as sluice retaining walls, reservoirs, and storage tanks, can also develop thermal gradients that result in cracking due to their significant thickness and the considerable external restriction on their ability to free deformation.

The risk of cracking is generally assessed by comparing the tensile strength of concrete with the thermal stresses induced in mass concrete structures [4]. Additionally, crack criteria can be based on strain analysis and the concrete’s strain capacity [5]. The primary cause of these cracks is inhomogeneous volume changes due to thermal and moisture gradients during the cement hydration process [20], whose exothermic nature generates significant temperatures in early-age concrete structures. It needs to be highlighted that the composition of the concrete mix significantly affects the exothermic nature of cement hydration and the subsequent temperature rise. Different types of cement have varying heat generation characteristics, with ordinary Portland cement (OPC) typically generating more heat compared to blended cement with slag or fly ash. The cement content in the mix also plays a crucial role; higher cement content increases the amount of heat generated during hydration as more cement particles react with water. Further, the type of aggregate affects the thermal properties of the mix, where aggregates with high thermal conductivity can help dissipate heat more effectively. Consequently, the overall proportions of cement, water, aggregates, and admixtures in the concrete mix determine its thermal behavior. Optimizing these factors within the mix composition is essential for managing the heat generated during hydration and preventing early-age thermal cracking in mass concrete structures. Another important task is selecting the composition of the concrete mixture to achieve the desired mechanical properties and durability of hydrated cement [21]. It is worth noting that the impact of the concrete mix composition on the development of hydration-induced temperature increases has been extensively studied, with detailed findings available in research works [22,23,24,25,26].

As previously mentioned, the issue of heat released during hydration and the resulting temperature increase primarily occurs in structures with large cross-sectional dimensions. While thin sections of structural members dissipate heat quickly, thicker sections experience conditions close to adiabatic, with maximum temperatures reaching 50–70 °C [24,27]. Moreover, the internal temperature decreases slowly, while the surfaces in direct contact with the environment cool rapidly, creating thermal gradients across the sections of concrete members.

Apart from the heat generated during the hydration process, a significant cause of high temperature increases and their uneven distribution between the surface layers and inner parts of massive concrete members is the low thermal conductivity of concrete, which slows the natural cooling process. Therefore, assessing the risk of cracking based on stress or strain criteria must be preceded by determining thermal loads. This task is complex because the source of these loads is the concrete itself. These non-mechanical thermal loads depend on various technological and material factors [20,22,23,28,29], such as concrete mix proportions and types of components, conditions during concreting and curing (e.g., the initial temperature of concrete, the formwork type, and the use of insulation or pipe cooling), concreting technology (e.g., segmental concreting), and boundary conditions (e.g., the ambient temperature, the temperature of neighboring members, wind, and humidity). Additionally, the dimensions of the analyzed element, particularly the cross-section dimensions, largely determine the level of temperature increase during the hardening process.

It is widely recognized that thermal loads induced by the hydration process can be hazardous in mass concrete. However, the definition of mass concrete is quite broad. Typically, mass concrete is defined as any volume of concrete with dimensions so large that measures must be taken to reduce the risk of cracking due to volume changes from the heat generated during hydration [27]. Simultaneously, there is no universally recognized and strict method for assessing a structure’s susceptibility to early hydration-induced effects. However, several common simplified methods are used for the initial classification of massive structures. These methods typically focus on basic geometric and material properties to categorize structures based on their potential thermal behavior. For instance, structures with cross-sectional dimensions greater than 1 m are often expected to experience significant thermal gradients due to their size. A high volume-to-surface area ratio also indicates a massive structure, as the core can retain heat longer than the surface can dissipate it. Additionally, high cement content or the use of cement without supplementary cementitious materials (SCMs) may classify a structure as massive due to the increased heat generated during hydration. Experience shows that mass concrete structures often appear in construction, including previously mentioned large water structures like dams, massive foundation slabs, bridge spans and supports, tanks for liquids and bulk materials, walls in nuclear power plants, and supports for tall buildings. Additionally, there are numerous examples of cracks in structures of a relatively small thickness but with very limited freedom for early thermal-shrinkage deformations, leading to severe cracking [2,10,30,31,32,33]. Consequently, the lack of a universally recognized strict definition of a massive structure can cause confusion in the practical assessment of mass concrete and the need to predict the potential cracking risk.

Simultaneously, predicting and considering these effects during the design process is essential to minimize the risk of cracking and the crack width. The main challenge arises from the unique nature of early-age loads, which originate from the material itself. These loads are generated during concrete curing, while the material gains strength and elastic properties as hydration progresses. The magnitude of early-age thermal loads is influenced by various technological and material factors, including curing conditions, construction technology, concrete mix composition, and structure dimensions as mentioned before. In addition to the critical role of concrete mix composition discussed earlier, construction techniques are equally vital. For instance, the method of concrete placement and curing practices significantly affects heat distribution and temperature rise, with cooling techniques such as embedded pipes helping to manage temperatures [34,35]. Environmental conditions, such as ambient temperature, wind, and humidity, also impact the rate at which heat dissipates from the concrete surface [5]. Furthermore, the type of formwork and insulation employed, along with the size and shape of the structure, influence heat retention and distribution, with large cross-sections and intricate shapes particularly prone to uneven thermal gradients.

Further, considering the material and technological factors, it is important to note that effective methods for reducing thermal stress and the risk of cracking in mass concrete structures directly involve these factors. The most common approach is to optimize the concrete mix design by using low-heat cement or incorporating supplementary cementitious materials (SCMs) like fly ash or slag, which lower the heat generated during hydration. Construction practices such as staged pouring and the use of cooling techniques such as embedded cooling pipes or chilled aggregates in the concrete mix can also mitigate temperature increases. Additionally, proper curing methods, such as wet curing or applying insulating materials on exposed surfaces, help regulate internal temperature gradients and minimize thermal stress. It should be underlined that only by integrating both material and technological methods, crack-free mass concrete structures can effectively withstand thermal variations.

Currently, there is a general tendency to use finite element (FE) modeling for most civil engineering problems. This method can also be applied to design mass concrete members subjected to early-age effects [12,24,36,37]. The FE method allows designers to perform comprehensive analyses, including temperature, moisture, and stress development in the member during concrete hardening, with specified material properties and technological conditions. Some guidelines even recommend using numerical methods for designing such structures. However, numerical methods require specialized software, which is not always accessible to structural engineers [38]. Additionally, the amount of data needed for numerical analysis requires knowledge of thermo-mechanics and often exceeds typical engineering knowledge. Further, assuming the appropriate data poses many difficulties. These data can be obtained through experimental tests of the heat of hydration, as well as the thermal and mechanical properties of concrete, and archival data, such as information on environmental conditions during concreting. However, in the typical activities of engineers in design offices, such an approach is not always feasible. Therefore, in many cases, recommended data or proper analytical formulas are used to determine the necessary inputs for the FEA model concerning thermal and mechanical properties [38]. It should be noted that these input data significantly impact the results obtained from the FEA model, as demonstrated in [37]. Therefore, simple analytical methods are still applied in this area [39]. The major advantage of analytical methods is their ease of use and applicability without specialized software. However, these methods provide only rough estimations due to numerous simplifications and are usually limited to structures with typical shapes, simple technological conditions, and standard support conditions.

Given the complexities outlined above, it is advisable to propose a systematic approach to determine when advanced FE modeling is necessary, which requires significant time and resources, and when simpler methods can be initially employed. As mentioned, the current definition of mass concrete structures—those susceptible to adverse effects like cracking due to temperature increases from hydration heat—is imprecise. Therefore, the classification of a structure as “mass concrete” should be the starting point in practice. Once a structure is classified as mass concrete, designers need to assess the potential temperature rise and resulting thermal stresses or strains. Simple analytical methods can provide initial estimates of these temperatures and stresses/strains. For example, empirical formulas or semi-empirical methods can offer quick insights into expected temperature profiles and stress/strain distributions. These methods might include simplified heat flow models that consider concrete’s low thermal conductivity and the resulting temperature gradients. If the simplified analysis indicates a potential risk of cracking, more detailed numerical methods, such as finite element analysis (FEA), may be employed. FEA allows for a more precise evaluation of thermal and moisture gradients, considering the complex interactions between the hydration heat, environmental conditions, and structural constraints. It can model the temperature distribution across the structure and predict the development of thermal stresses over time. Additionally, FEA can incorporate various material properties and boundary conditions, providing a comprehensive understanding of the structural behavior during the early-age phase. This detailed analysis assists in designing appropriate measures to mitigate the risk of cracking, such as optimizing the concrete mix, controlling the curing conditions, or implementing cooling strategies like pipe cooling. Therefore, the decision to use advanced FE modeling should be based on the outcomes of initial simplified assessments, ensuring the efficient use of resources while maintaining structural integrity.

To address these challenges, a three-step method for evaluating structures in the context of early-age thermal effects is proposed. The first step involves a simplified method to classify structures as massive based on their dimensions and expected thermal behavior. The second step entails estimating hardening temperatures and levels of thermal stress using straightforward analytical techniques. The third step, reserved for structures identified as having a potential risk of early thermal cracks, involves detailed numerical modeling. To illustrate the application of this three-step method, an exemplary analysis of a mass concrete wall constructed on a foundation is presented. This proposed three-step method—classification, simplified analysis, and detailed numerical modeling—offers a structured approach to evaluate and mitigate early-age thermal effects.

## 2. Proposal of Multi-Step Procedure

### 2.1. Step 1–Preliminary Assessment of Structure

As mentioned before, ACI 116R [40] defines mass concrete, stating that it is “any volume of concrete with dimensions large enough to require the measures be taken to cope with generation of heat from the hydration of the cement and attendant volume change, to minimize cracking”. Another definition, given by K. Flaga [41] involves the use of the surface modulus to define massive structures. This modulus can be calculated as follows:

(1) $$m_{s}=\frac{S}{V}$$

where S—the surface area, $m^{2}$; V—the volume of the element, $m^{3}$.

The classification based on the surface modulus and self-heating temperature categorized concrete structures into three types: massive, medium–massive, and non-massive. Massive structures have a surface modulus ($m_{s}$) of less than 2 and experience a self-heating temperature rise of more than 20 °C. Medium–massive structures exhibit a surface modulus ranging from 2 to 15 and a self-heating temperature between 3 °C and 20 °C. Non-massive structures, on the other hand, have a surface modulus greater than 15 and a self-heating temperature increase of only 1 °C to 3 °C.

The concept mentioned above solely concerns the geometry of the element. However, it is widely acknowledged that there are several other factors that significantly impact the risk of thermal cracking in massive concrete structures, regardless of the geometry that undoubtedly governs heat dissipation. Some of the most influential factors include the type and content of the binder, as well as the temperature during casting and curing. Therefore, recent modifications in the concept of massivity take into account these effects, resulting in an improved massivity index [42]:

(2) $$m_{cor}=\frac{m_{s}}{k_{f}\times k_{b}\times k_{∆T}}$$

where $k_{f},k_{b},k_{∆T}$—correction factors accounting for the cement type, the binder content, and the temperature differential, respectively. The factor $k_{f}$ takes into account the potential heat produced by the binder used. According to this proposal, this factor measures the reduction in heat compared to a reference value of CEM I when blended cements are used. In other words, it represents the ratio of the heat output of blended cement with supplementary cementitious materials (SCMs) to that of pure Portland cement-CEM I:

(3) $$k_{f}=\frac{Q_{SCM}}{Q_{CEMi}}$$

The proposed values of the relative heat factor ($k_{f}$) for different cement types reveal significant variations based on the composition of the cement [42]. The ordinary Portland cement, CEM I 42.5, serves as the baseline with a relative heat factor of 1.00 and a heat evolution of 366 J/g at 72 h in a semi-adiabatic test. When fly ash (FA) is added to the cement, the relative heat factor decreases progressively: with 10% FA, *k_f_* is 0.89 (325 J/g); with 30% FA, $k_{f}$ drops to 0.55 (200 J/g); and with 50% FA, it further reduces to 0.31 (112 J/g). Similarly, the inclusion of ground granulated blast furnace slag (GGBS) also reduces the relative heat factor: 10% GGBS results in a $k_{f}$ of 0.91 (334 J/g); 30% GGBS reduces it to 0.70 (255 J/g); 50% GGBS leads to a $k_{f}$ of 0.63 (232 J/g); and 70% GGBS brings it down to 0.43 (157 J/g). These values consider that both FA and GGBS additions decrease the heat evolved during the curing process, with higher percentages of these supplementary cementitious materials leading to lower relative heat factors.

The next factor, $k_{b}$, is a correction factor that depends on the binder content. It can be calculated by dividing the binder content in the mix by 300 kg/m^3^:

(4) $$k_{b}=\frac{binder\;content,\;kg/m^{3}}{300\;kg/m^{3}}$$

Finally, the correction factor for temperature differential, $k_{∆T}$, can be determined using the following equation:

(5) $$k_{∆T}=\frac{T_{fresh}-T_{ambient}+T_{adi,rise}}{T_{adi,rise}}$$

where $T_{fresh}$—the fresh concrete temperature, $T_{ambient}$—the expected ambient temperature at the time of maximum concrete temperature, and $T_{adi,rise}$—temperature increase due to hydration under adiabatic conditions.

Therefore, this stage of the analysis should conclude with a determination of whether a more detailed assessment of the temperature and potential cracking of the structure is warranted.

### 2.2. Step 2–Simple Analytical Method

If in step 1 the structure was identified as massive with an initially predicted high self-heating temperature, this prediction can be verified in the next step using a straightforward analytical method.

The proposed method facilitates the simple determination of temperature rise and strains in walls during concrete hardening. It is important to note that a similar method for evaluating temperatures in early-age mass foundation slabs has already been presented [39]. The method for determining temperatures was developed and validated based on extensive numerical analyses of concrete walls [43]. The evaluation of the cracking risk is generally based on Eurocode [44,45] and CIRIA C766 [5] guidelines. The hardening temperatures can be assessed through the following procedure:

(a) Calculating the self-heating temperature of concrete under adiabatic conditions (without any heat exchange with the environment) using the following expression:

(6) $${∆T}^{adiab}=\frac{Ca_{Q}Q_{\infty }}{c_{b}\rho_{b}}$$

where

C—the cement content in concrete, kg/m^3^;

$a_{Q}$—a coefficient that represents the heat generated during the early stages of concrete hardening, influenced by the rate of heat generation determined by the mineral composition of the cement or technical specifications. Table 1 provides typical values for this coefficient. When using concrete with Portland cement CEM I blended with slag and/or fly ash, the coefficient can be determined based on the proportion of Portland cement, slag, and fly ash in the binder.

$Q_{\infty }$—the total heat released during cement hydration, measured in kJ/kg, can be determined by considering the mineral composition of the cement. Table 2 provides indicative values of this total heat for typical cements.

$c_{b}$—the specific heat of concrete, kJ/(kg∙°C), depending mainly on the aggregate type,

$\rho_{b}$—the volume density of concrete, kg/m^3^.

(b) Calculating the self-heating temperature involves adjusting for the reduction coefficient χ, which accounts for heat exchange with the surroundings under conditions that are not fully adiabatic:

(7) $${∆T}_{red}^{adiab}={∆T}^{adiab}\chi$$

The coefficient χ equals 1 under strictly adiabatic conditions, and less than 1 otherwise. Recommended values of this coefficient, tailored to the wall thickness and derived from the numerical analysis [43], are detailed in Table 3 and Table 4.

(c) Calculating the internal temperature $T_{int}$ of the element, according to the following formula:

(8) $$T_{int}=T_{bo}+{∆T}_{red}^{adiab}$$

where

$T_{bo}$ is the initial concrete temperature.

**Figure 1:**
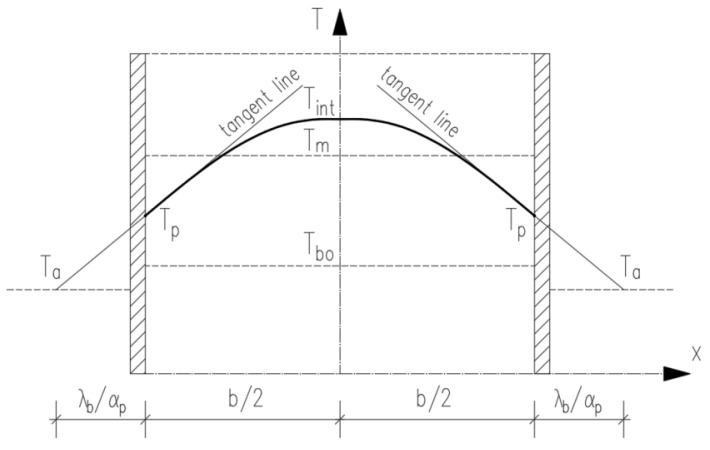
Distribution of the temperature in the wall cross-section.

(d) To determine the temperature at the wall surface, $T_{p}$ (Figure 1), one considers the surface temperature gradient, ${\left.\frac{dT\left(\tau \right)}{dx}\right|}_{p}$, along with the heat conduction coefficient, $\lambda_{b}$, the heat transfer coefficient, and $\alpha_{p}$, the ambient temperature, $T_{a}$, and applies the third boundary condition:
  (9) $${\left.\frac{dT\left(\tau \right)}{dx}\right|}_{p}=\frac{\alpha_{p}}{\lambda_{b}}\left(T_{p}-T_{a}\right)$$

Given a parabolic temperature distribution across the cross-section of the element (Figure 1), we can express $T_{p}$ as follows:

(10) $$T_{p}=T_{int}+\frac{T_{a}-T_{int}}{\frac{b}{2}+2\frac{\lambda_{b}}{\alpha_{p}}}\cdot \frac{b}{2}$$

where b is the wall thickness.

Calculating the average temperature throughout the thickness of the wall and assuming a parabolic temperature distribution across the element, the average temperature can be estimated using the following formula:

(11) $$T_{m}=T_{int}-\frac{1}{3}\left(T_{int}-T_{p}\right)$$

It is crucial to note that when dealing with wall structures cast on an existing foundation, the difference ∆T between the maximum self-heating temperature and the ambient temperature during the cooling phase is of primary importance. This significance stems from the tensile restraint strains and stresses that occur as the wall cools. Moreover, for walls of considerable thickness, the temperature disparity between the interior and the surface, $∆T_{1},$ may also play a role in determining self-induced strains and stresses. Finally, both values are calculated as follows:

(12) $$∆T=T_{m}-T_{a}$$

(13) $$∆T_{1}=T_{int}-T_{p}$$

Knowing the key temperature values allows you to determine deformation and the potential risk of cracking. Hence, the restraint tensile strain, $\varepsilon_{r}$, is calculated from the following formula:

(14) $$\varepsilon_{r}=K_{1}R\alpha_{T}\Delta T$$

Potential self-induced strains can be calculated in a similar way:

(15) $$\varepsilon_{r1}=K_{1}R\alpha_{T}\Delta T_{1}$$

where

**Figure 2:**
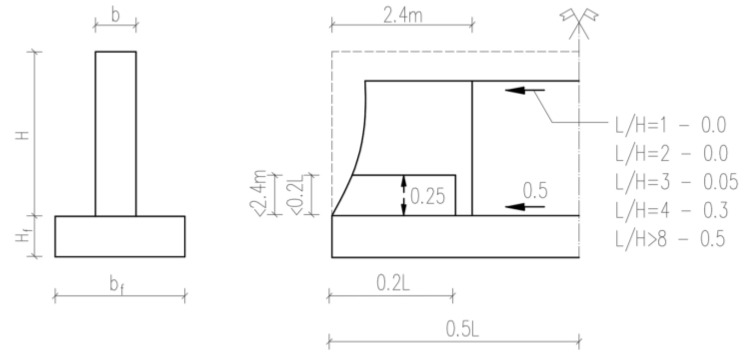
The restraint factor, R, for a wall with limited deformation freedom along the lower edge [45].

$\alpha_{T}$—the coefficient of thermal expansion for concrete, dependent on the type of aggregate; the recommended values are listed in Table 5;

$K_{1}$—the coefficient of stress relaxation due to creep under sustained loading; the recommended value is $K_{1}=0.65$ or 1.0 when the R factor is taken based on [45];

R—the restraint factor reflecting the degree of limiting deformation freedom. In the case of walls cast on the existing foundation, R may be assumed according to [5] or based on equations enclosed in ACI [4]. Values of the R factor corresponding to the simplest case of a wall with limited deformation freedom along the lower edge are presented in Figure 2. For self-induced strains, CIRIA C766 recommends R=0.42.

The risk of cracking is evaluated by comparing the tensile strains, $\varepsilon_{r}\;$or $\varepsilon_{r1}$, induced in the wall structure with the corresponding ultimate strains, $\varepsilon_{ctu}$. Therefore, cracking is likely to occur when the following condition is met:

(16) $$\varepsilon_{r}>\varepsilon_{ctu}\;\mathrm{or}\;\varepsilon_{r1}>\varepsilon_{ctu}$$

The ultimate strains, $\varepsilon_{ctu}$, can be calculated based on [5], for concrete class C30/37 with various types of aggregate (Table 6). When the concrete class differs from class C30/37, the values given in Table 6 should be recalculated according to the following formula:

(17) $$\varepsilon_{ctu}=\varepsilon_{ctu_{C}30/37}\left[0.63+\left(f_{ck,cube}/100\right)\right]$$

It should be emphasized that the analytical model simplifies the assessment to only determine the risk of cracking. This simplification is significant for several reasons. Firstly, we only consider the maximum temperature values in the cross-section without analyzing their variation along the height of the wall. Furthermore, based on these temperatures and the distribution of the restraint coefficient according to the EC2 standard (Figure 2), the analytical model estimates that maximum tensile strain will occur at the point of contact between the wall and the foundation or the previous stage of concreting. This distribution is not precise, as evidenced by previous publications [32,43,46] and the FEA analysis results are presented in the next section. While it is possible to use the distribution of the restraint coefficient along the height of the wall to determine the distribution of tensile strains and specify the extent of cracking at the height of the wall, this method remains approximate. Additionally, the analytical model considers average temperatures across the wall thickness, thus it does not analyze the development of surface cracks, which usually appear first when early stripping is performed. Nevertheless, the presented analytical method provides a relatively quick assessment of temperature increase and the risk of cracking. Another aspect of the proposed method that could be refined is the introduction of additional safety factors and strain limits for a wall’s cross-section depending on the exposure class, as stated in the Swedish guidelines [47], whose validity has been confirmed in [11].

Finally, this stage of the analysis may confirm the potential risk of cracking and indicate the need for a more detailed assessment of the structure, which can be conducted using the finite element (FE) method.

### 2.3. Step 3–FE Analysis

The results of step 2, if they indicate high temperatures and a risk of cracking, may justify conducting a more detailed analysis. It is especially valid for more complex structures where it may be necessary to perform numerical analysis to achieve accurate results. In such cases, the Finite Element Method (FEM) is commonly used. The application of FEM requires a specialized computational tool that can simulate early-age concrete phenomena. These tools, depending on the mathematical model used, can identify thermal–mechanical or thermal–moisture–mechanical phenomena and assess the risk of cracking. In [38], an evaluation of commercial software tools designed to predict these effects is provided. The next section outlines the general analysis methodology using the DIANA FEA 10.6 software.

**Figure 3:**
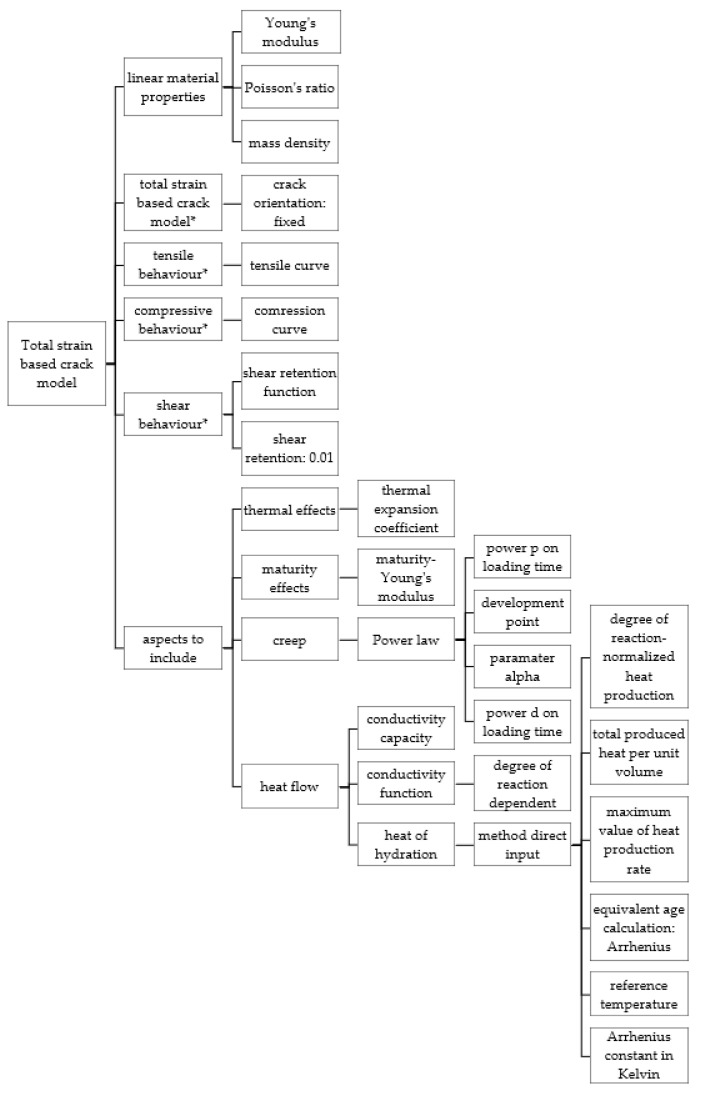
The path of the selection of aspects to include in the “Total strain-based crack model” for concrete. * Aspects not included in analysis performed in Section 3.3.

**Figure 4:**
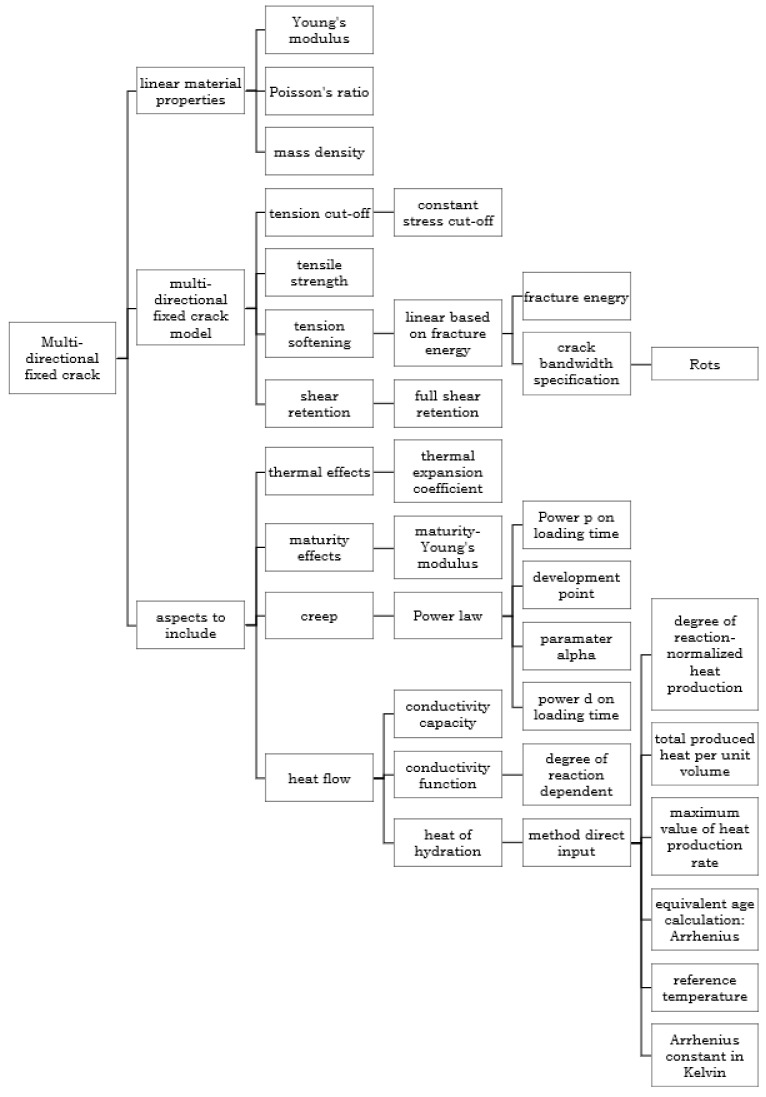
The path of the selection of aspects to include in the “Multi-directional fixed crack model” for concrete.

The model is created by generating a finite element mesh of a defined geometry. This geometry can be either imported as an externally defined model or created within the DIANA preprocessor. Defining the geometry shape involves assigning an element class, which includes various types of elements such as two-dimensional body elements, three-dimensional bodies or solid plates and shells, trusses and cables, beams, interfaces, springs and mass elements, reinforcements, and more. Additionally, the material model needs to be assigned. There are various material models available for concrete and masonry, soil, steel, composites, etc. DIANA provides a wide range of material models, for both structural and flow analysis. The software allows for the selection of different aspects of the material properties, including thermal effects, maturity effects, creep, shrinkage, heat flow, Rayleigh damping, additional dynamic 2D line mass, additional dynamic surface mass, and additional dynamic 3D line mass. In the example outlined in Section 3.3, two material models were utilized for the concrete in the analyzed wall and foundation slab: the “Total strain-based crack model”, which considers the viscoelastic behavior of the concrete without cracking, and the “Multi-directional fixed crack” model, which accounts for cracking. Furthermore, the “Linear elastic isotropic” model was employed for the lean concrete. Each material model requires inputting a specific number of parameters for calculations. Figure 3 and Figure 4 illustrate the process of selecting the different aspects to include in the analysis when applying a particular material model.

For the reader’s convenience in the use of the discussed algorithm, the original notations from DIANA software are preserved along with explanations of their corresponding values. The temperature field may be determined based on the partial difference equation for energy balance:

(18) $$\rho_{c}c_{0}\frac{\partial T}{\partial t}=k\left(\frac{\partial^{2}T}{{dx}^{2}}+\frac{\partial^{2}T}{{dy}^{2}}+\frac{\partial^{2}T}{{dz}^{2}}\right)+q_{v}$$

with T—thetemperature, $\rho_{c}$—the density of concrete, $c_{0}$—specific heat, t—time, k—the thermal conductivity, x,y,z—coordinates, and $q_{v}$—the rate of internal heat generation calculated according to the following formula:

(19) $$q_{v}=Af(\alpha )e^{-\frac{E_{a}}{R_{v}T}}$$

with $f\left(\alpha \right)$—the normalized heat generation rate, A—the rate constant, $R_{v}$—the ideal gas constant ($R_{v}$= 8.314 J/(mol·K)), $E_{a}$—the apparent activation energy, and α—the degree of hydration.

The degree of hydration serves as an indicator to assess the advancement of the cement hydration process. It spans from 0, which represents the initial addition of water to the cement, to 1, which signifies the completion of hydration. In this particular approach, the hydration degree α is defined as the ratio between the heat released by the cement up to a specific point in time and the total heat $Q_{max}$ expected to be released upon complete hydration. $Q_{max}$ can be determined through experimental means, such as isothermal calorimetry. As stated by [48], when two concrete samples exhibit an equal degree of heat evolution, they are considered to have the same equivalent age, as described by the following equation:

(20) $$t_{eq}=\int_{0}^{t}e^{\frac{E_{a}}{R_{v}}\left(\frac{1}{T(\tau )}-\frac{1}{T_{ref}}\right)}d\tau$$

where $T_{ref}$—the constant temperature subjected to concrete and T(τ)—the generic temperature history subjected to concrete.

In the context of heat flow analysis, the heat of hydration, conductivity, and heat capacity of the material can be specified. These parameters can either remain constant or vary with temperature (T) and the degree of reaction. In the case of properties that are dependent on the degree of reaction, it is important to provide a diagram illustrating a range of degrees of reaction and their corresponding values for each property. During the analysis, DIANA utilizes linear interpolation to determine the current value of the material properties. The convective heat transfer between the concrete and its surrounding environment is taken into account through a coefficient $h_{cr}$ that considers both convection and radiation. This coefficient simplifies the FEA simulation of concrete boundaries [48]. In order to determine the heat transfer from the outer surfaces, the following boundary condition is applied:

(21) $$\tilde{q}=h_{cr}\left(T_{surf}-T_{a}\right)$$

with $\tilde{q}$—the heat transfer, $h_{cr}$—the convection-radiation heat transfer coefficient, $T_{surf}$—the temperature of the boundary surface of the element, and $T_{a}$—the environmental temperature. It is important to note that the equation mentioned above is applicable only to the typical range of temperature differences between concrete surfaces and the environment. In practice, there are instances where certain surfaces are not in direct contact with the air due to the presence of formwork or cover application. To better simulate the system of individual material layers that are connected in series, an equivalent heat transfer coefficient $h_{eq}$ is often defined. This coefficient can be calculated using the following method:

(22) $$h_{eq}={\left(\frac{1}{h_{cr}}+\sum_{1}^{n}\frac{L_{i}}{k_{i}}\right)}^{-1}$$

where $L_{i}$—the material thickness of *i*-th layer and $k_{i}$—the thermal conductivity corresponding to the *i*-th layer.

Furthermore, it is necessary to describe the viscoelastic behavior. To achieve this, a creep function $J\left(t,t_{0}\right)$ can be used. DIANA offers two examples of creep functions: the Kelvin Chain model and the Double Power law. The relationship between stresses and strains can be expressed as follows:

(23) $$\varepsilon \left(t\right)=\int_{-\infty }^{t}J\left(t,t_{0}\right)\bar{C}\dot{\sigma }(t_{0})dt_{0}$$

where $\bar{C}$—the dimensionless matrix, as a function of Poisson’s ratio,

The software offers users a variety of analysis types to choose from, including linear static analysis, dynamic analysis, Euler stability analysis, potential flow analysis, coupled flow-stress analysis, phased analysis, and parameter estimation. For nonlinear analysis, the software provides several material models such as plasticity, viscoplasticity, cracking, creep, hyperelasticity, liquefaction of soil, interface nonlinearities, and others. Additionally, users can specify the time dependency of temperature development, concentration, and maturity. The phased analysis feature allows for the modeling of phased construction, which helps determine the effects of construction history and highlights critical construction stages.

**Figure 5:**
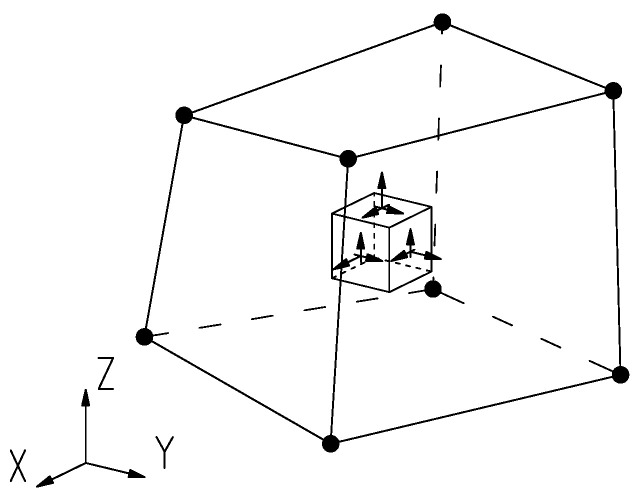
Characteristics of solid elements.

In the analysis of voluminous structures, such as massive structures, the utilization of solid elements is pertinent (Figure 5). These elements possess distinct qualities, including three-dimensional stress, arbitrary loading, and dimensions in three axial directions X, Y, and Z, all of which are on a similar scale.

**Figure 6:**
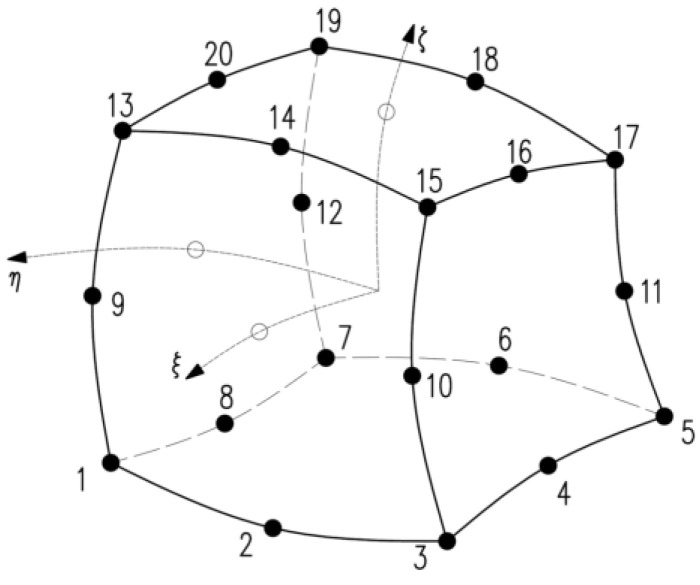
Twenty-node solid brick element.

DIANA uses various types of three-dimensional structural elements, including tetrahedrons, pentahedrons (pyramids), hexahedrons (wedges), and octahedrons (bricks). These elements differ in shape and the number of nodes they have. The displacement field can be interpolated linearly, quadratically, or cubically. Linear interpolation functions have two nodes at each edge, quadratic interpolation functions have three nodes at each edge, and cubic interpolation functions have four nodes along an edge. In the FEA described in Section 3.3, twenty-node solid brick elements were utilized, as depicted in Figure 6.

Support sets are utilized in geometry to establish fixed translations and rotations in various directions. They can be attached to solids, faces, lines, and points. Moreover, different coordinate systems can be employed.

Geometric shapes can be subject to two primary types of loads. The first type is the global load, encompassing dead weight, equivalent acceleration, centrifugal load, and base excitation. These loads are applied to the entire element. The second type of load is specific to particular shapes, faces, lines, or points. Examples of this load type include distributed force, prescribed deformation, pore pressure, hydrostatic pressure, modal pushover, incremental temperature load, and more. These loads can exhibit either time or frequency dependence. The available load types are contingent upon the shape type and the project settings for analysis, such as structural, heat flow, groundwater flow, and fluid–structure interactions. They can be applied in either global or normal directions.

## 3. Example of Application

**Figure 7:**
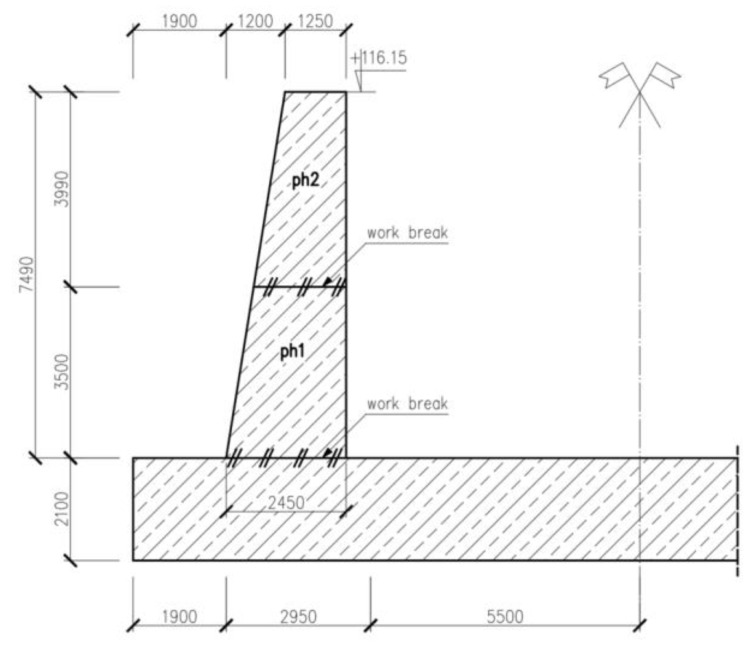
The geometry of the analyzed wall.

The reference case presented in the following section pertains to a wall of the sluice structure. Given the considerable length of the sluice (approximately 180 m), it was divided into several segments. The focus of this analysis is specifically on one of the wall segments, which measures 10.35 m in length, 7.49 m in height, and has a variable thickness that ranges from 2.45 m at the bottom to 1.25 m at the top. The foundation for this wall consists of a slab with a thickness of 2.1 m. Figure 7 presents a depiction of the wall’s geometry. It is worth noting that due to its considerable thickness, the analyzed wall was further divided into two stages of concreting, approximately halfway up its height. The composition of the concrete mix used for the structure is shown in Table 7.

### 3.1. Step 1

The preliminary assessment of the potential for thermal cracking effects during the pre-design stages, which requires more detailed studies, can be conducted using simplified approaches such as the massivity index, also known as the surface modulus $m_{s}$ (described in Section 2.2). For the specific case study presented here, with wall dimensions of 7.4 × 10.35 × 1.25 ÷ 2.45 m³, the surface modulus was calculated by dividing the exposed area (excluding the bottom surface in contact with the ground) by the volume (Equation (1)). Under these conditions, the value of $m_{s}$ was calculated to be 1.44 /m, which falls below the threshold of 2.0 /m. Therefore, the structure is considered to be massive, with a predominant impact of thermal strains and close-to-adiabatic conditions in the core [41]. To further refine the surface modulus approach [42], adjustments can be made by considering corrective factors, as outlined in Equation (2). These factors take into account the amount of cement used, the presence of supplementary cementitious materials, the expected adiabatic temperature rise, as well as the environmental and mixture temperatures. The initial massivity value of 1.44 /m was corrected by dividing it by the following three factors:

Factor $k_{f},$ to consider the presence of supplementary materials in the binder, calculated from Equation (3) for $Q_{SCM}$ = 208.23 J/g (determined experimentally for CEM III/A 42,5N-HSR/NA), which resulted in $k_{f}$= 0.57;

Factor $k_{b},$ to take into account the actual binder content in the mix, (which was 370 kg/m^3^, resulting in $k_{b}$ = 1.233;

Factor $k_{∆T},$ which depends on the temperature of the fresh mixture ($T_{fresh}$ = 20 °C), the average ambient temperature ($T_{ambient}$ = 10 °C) and the adiabatic temperature rise expected ($T_{adi,rise}$= 42 °C, as available from experimental data [42]), thus resulting in $k_{∆T}$ = 1.238.

The obtained corrected massivity factor $m_{cor}$ was 1.66 /m, which is still below 2 /m despite using the slag cement. Therefore, it remained necessary to conduct a detailed thermal study of this structure, as described in this paper.

### 3.2. Step 2

At this stage, the calculations for the hardening temperature and thermal deformations were performed according to step 2 of the proposed thermal analysis for massive structures. These calculations were conducted for two stages of concreting the wall shown in Figure 7. The data used in the calculation are listed in Table 8 while the results are summarized in Table 9 for the reader’s convenience. The estimated temperatures indicate significant self-heating of the relatively thick wall. This is important because, in such cases, both internal constraints and the temperature differences—between the interior and surface of the wall, as well as between the maximum and the final stable wall temperatures—are significant for cracking risk.

In detail, in both wall segments, there was a significant increase in the self-heating temperature of the concrete due to the released heat of hydration. In the first segment, with a height of 3.5 m and an average thickness of 2.17 m, a simplified method estimated an increase in temperature of 39.17 °C from the initial concrete temperature. In the second segment, with a height of 3.99 m and an average thickness of 1.57 m, the increase was 34.81 °C. These temperatures are much higher than 20 °C (see Section 2.1), indicating that the analyzed wall is a mass concrete structure with a risk of early thermal cracks. Additionally, the wall is subject to both external and internal restraints. External restraints arise from the wall’s connection to the previously concreted slab (stage 1) and the lower segment (stage 2 restrained at stage 1). Internal restraints result from the temperature difference between the inside of the wall and its surface. The relevant temperatures for strain analysis are 35.49 °C and 33.05 °C (external restraint) and 26.04 °C and 20.29 °C (internal restraint) for stages 1 and 2, respectively.

The analysis of thermal crack risk confirmed a high likelihood of cracks, especially those caused by external restraint. The estimated tensile strains are 186 με and 174 με for the first and second stages of concreting, respectively. Although tensile strains due to internal constraints also exceed the strain capacity, the permissible values are only slightly surpassed. The relatively simple and quick calculations at this stage confirmed the risk of thermal cracks in the wall. Hence, in the absence of advanced tools for computer simulation of these effects, this stage can be used to further analyze potential methods for limiting temperatures or to calculate the reinforcement needed to control crack width.

### 3.3. Step 3

#### 3.3.1. Assumptions to Analysis

The massiveness of the wall structure confirmed in steps 1 and 2, along with the potential for high temperatures, induced the necessity for more detailed numerical analysis. Additionally, the complexity of constructing the wall in two stages and the interaction between external and internal restraints provided further impetus for this analysis.

**Figure 8:**
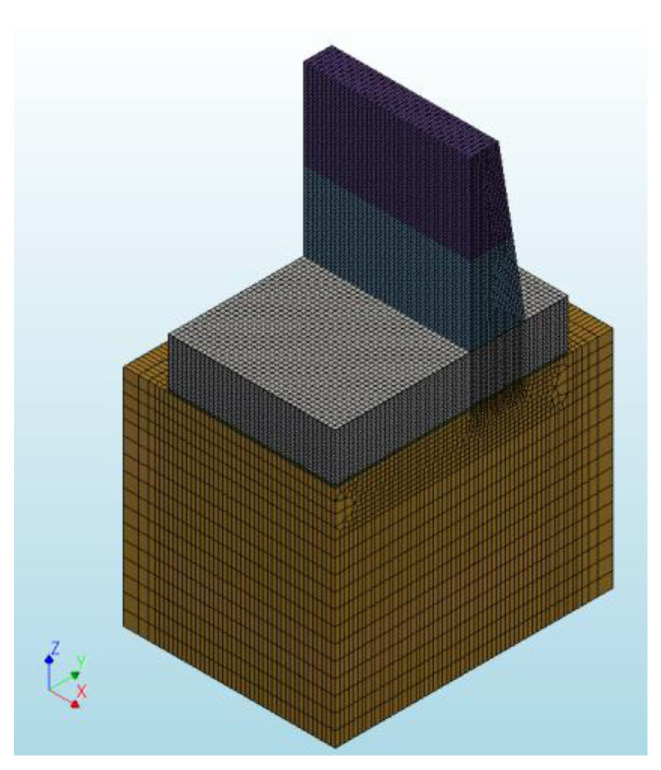
The geometry of the FE model.

In order to improve computational efficiency and simplify the model, the analysis was limited to only ¼ of the complete structure. The model, as illustrated in Figure 8, consists of the following components: the wall, which is the primary focus of the analysis, constructed in two distinct phases: phase 1 with a height of 3.5 m and phase 2 with a height of 3.99 m; the foundation slab, with dimensions of 7.4 × 10.35 m and a thickness of 2.1 m; a layer of lean concrete measuring 0.15 m beneath the foundation slab; the soil, which extends to a depth of 10 m (2 m beyond the plan view of the slab); and reinforcement for both the slab and the wall.

The material properties assumed in the study (Table 10) were taken based on data provided in the report from measurements. The remaining parameters were assumed according to the previously performed analysis for the massive foundation slab, presented in [37], supplemented with the data provided in [44,50]. It should be emphasized that, in terms of mechanical properties such as modulus of elasticity and tensile strength, the FEA considers the development of these properties during the hardening of the wall concrete and the influence of temperature on their development rate. The appropriate equation describing the results of the elastic modulus development tests is provided later in this chapter. The specified value of the elastic modulus for 28-day-old concrete in Table 10 is given as a reference to characterize mature concrete.

The analysis of early-age concrete members comprises two distinct stages. The initial stage involves simulating the temperature history resulting from cement hydration, while the subsequent stage entails evaluating the induced stresses. In this study, the calculation of thermal stresses was conducted by employing two concrete material models. The first model employed was the viscoelastic material model, which did not take into account crack development. The second model utilized was the “Multi-directional fixed crack” material model described in [51], which explicitly considers crack development. To accommodate this inclusion, the model necessitates the introduction of supplementary parameters, as outlined in Table 11.

Similarly to the modulus of elasticity, the FEA accounts for the development of strength over time and the influence of temperature on this development. The total impact of time and elevated temperature on the development of tensile strength with time is demonstrated by the equivalent time $t_{eq}$ (Equation (20)). The calculation of the rate of internal heat generation was performed using the formula from Equation (19), employing specific values obtained from individual tests carried out on cement CEM III/A 42,5N-HSR/NA, as demonstrated in Table 12.

Furthermore, the temperature-dependent Young’s modulus was applied. The evolving Young’s modulus is described by the following formula [52,53]:

(24) $$E\left(t\right)=\alpha_{1,E}e^{-{\left(\frac{\tau_{1,E}}{t_{eq}}\right)}^{\beta_{1,E}}}+\alpha_{2,E}e^{-{\left(\frac{\tau_{2,E}}{t_{eq}}\right)}^{\beta_{2,E}}}$$

where $\alpha_{1,E}$, $\alpha_{2,E}$, $\tau_{1,E}$, $\tau_{2,E}$, $\beta_{1,E}$, $\beta_{2,E}$—the parameters determined from measurements $\alpha_{1,E}=15$, $\alpha_{2,E}=20$, $\tau_{1,E}=2$, $\tau_{2,E}=4$, $\beta_{1,E}=1.5$, $\beta_{2,E}=1.5$, and $t_{eq}$—the equivalent age.

The Double Power Law model (Equation (25)) was used to consider the basic creep of concrete.

(25) $$J'\left(t,t_{0}\right)=\frac{1}{E\left(t_{0}\right)}+\frac{\phi_{1,DPL}}{E_{0}\left(t_{0}\right)}{\left(t_{0}\right)}^{-m_{DPL}}{\left({t-t}_{0}\right)}^{n_{DPL}}$$

The coefficients for this model were calculated based on the study referenced as [37]. In that study, the coefficients ($\phi_{1,DPL}=0.012$, $n_{DPL}=0.263$, and $m_{DPL}=0.016$) obtained for the concrete foundation slab were adjusted using the specific data provided in [3,54].

Shrinkage deformations were not taken into account because they generally have a negligible effect on stress values in massive structures [4,5,6].

Table 13 provides a list of the reinforcement used in the individual elements of the structure. The reinforcement was modeled using the embedded reinforcement type, and the Von Mises material model with parameters listed in Table 14 was applied.

Phased analysis was chosen to conduct the structural analysis. The duration of each phase is determined by the casting of the successive elements, as shown in Table 15. Furthermore, each phase is divided into a specific number of steps.

The phased analysis involved the transformation of the boundary conditions, depending on the advancement of the casting. The assumed values of the heat transfer coefficient $h_{cr}$ for each type of boundary condition are listed in Table 16.

**Figure 9:**
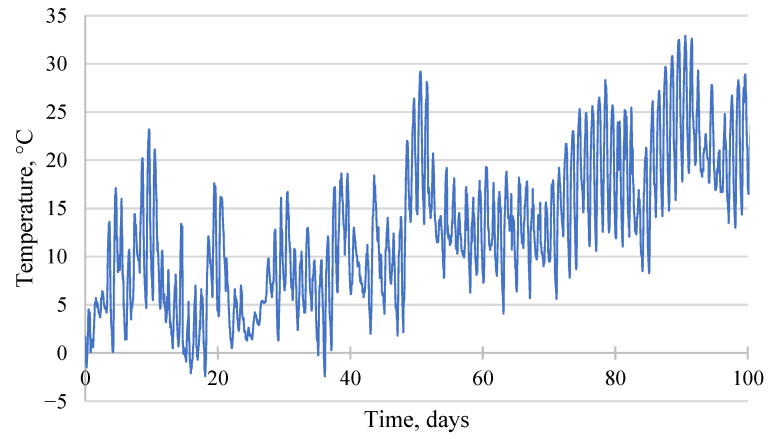
Ambient temperature.

The ambient temperature was assigned in a similar manner to step 2, taking into account the expected casting time during the spring season. However, to enhance the accuracy of the model, historical data recorded by the meteorological station in South Poland were utilized for this purpose (Figure 9). The initial temperature assigned to the particular elements of the model is listed in Table 18. The stress analysis was conducted for each phase of the process. The analysis took approximately 91 days to complete, starting from 22 March.

Twenty-node solid brick elements were assigned to the FE model. The assumed element sizes for the corresponding part of the structure are presented in Table 19.

#### 3.3.2. Results of Analysis

**Figure 10:**
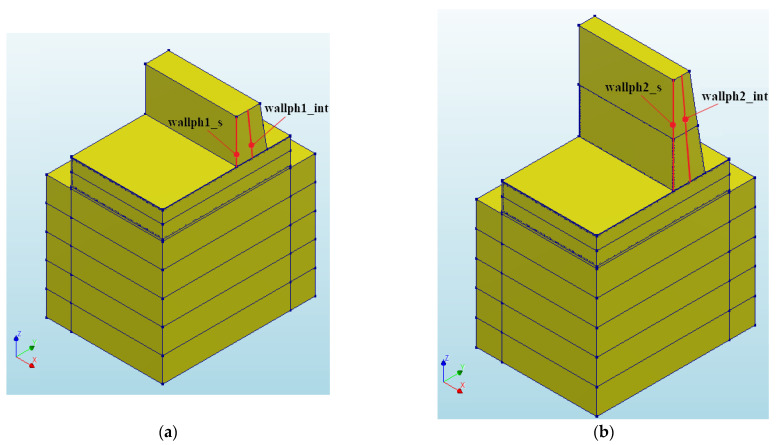
FE model presenting (**a**) the 1st phase of the wall, (**b**) the 2nd phase of the wall.

The results presented in the following subsection pertain to the 1st and 2nd phases of the wall, which were executed separately. The 1st phase of the wall was cast 28 days after the foundation slab was cast. Since the focus of this study is solely on the wall, the commencement of the wall casting was designated as “0” time in the subsequent figures. As illustrated in Table 15, the casting of the 2nd phase of the wall commenced 11 days after this time. The completed structure was then analyzed for a duration of 51 days. Considering that the maximum temperature and the temperature difference between the interior and surface are crucial factors for massive structures, the distribution of temperature and stress in two significant cross-sections of the wall were monitored. These sections were located in the center and on the surface of the wall, as depicted in Figure 10.

**Figure 11:**
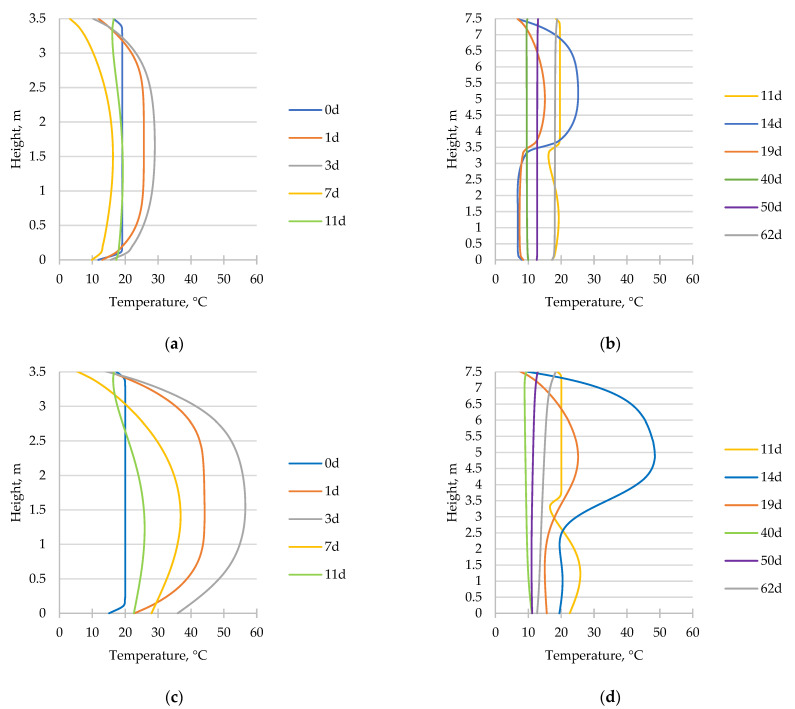
Distribution of temperature on the surface of the wall (**a**) wallph1_s, (**b**) wallph2_s, and in the middle cross-section of the wall (**c**) wallph1_int, and (**d**) wallph2_int, at selected moments of analysis, including 0 d—the time of the beginning of the analysis of the 1st phase of the wall (after 28 days from the casting of the slab); 11 d—the start of the casting of the 2nd part of the wall (after 11 days from the casting of the 1st phase); and 62 d—the end of the entire analysis.

Figure 11 displays the temperature distribution acquired for the 1st (a, b) and the 2nd (c, d) phases of the wall in two sections situated in the middle cross-section of the wall (wallph1_int, wallph2_int) and on the surface (wallph1_s, wallph2_s). The figures indicate that the maximum temperature and temperature difference between the interior and the surface of the structure for the 1st phase were both recorded 3 days after the casting, with values of 56.5 °C and 27.6 °C, respectively. Similarly, the extremum values for the 2nd phase were obtained 3 days after the completion of the casting (14 days from “0” time). The maximum temperature in the middle section was measured at 48.5 °C, with a temperature gradient of 23.5 °C.

**Figure 12:**
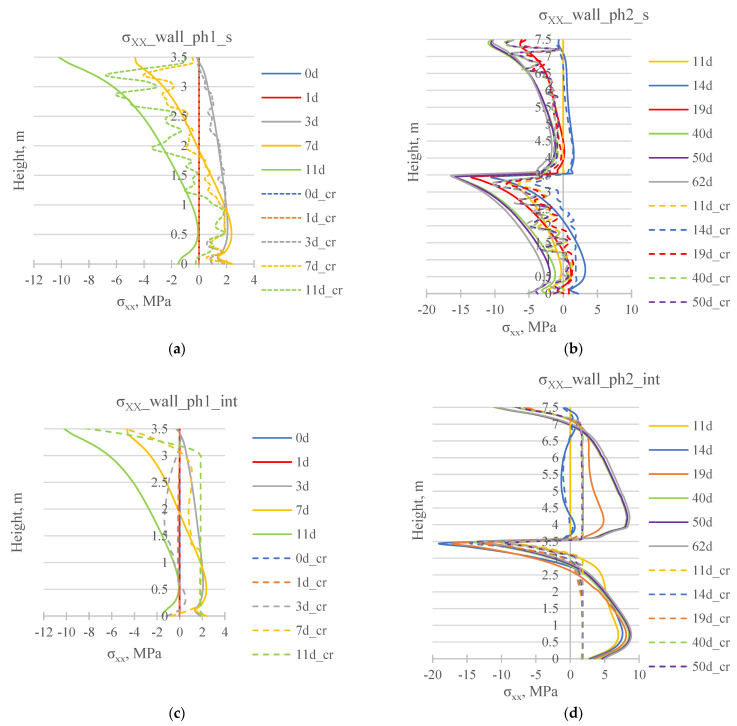
Distribution of stress $\sigma_{xx}$ on the surface of the wall (**a**) wallph1_s, (**b**) wallph2_s, and in the middle cross-section of the wall (**c**) wallph1_int, and (**d**) wallph2_int, at selected moments of analysis for the model without cracking consideration (solid line) and considering cracking (dashed line).

Figure 12 presents the comparison of the cross-sectional stress $\sigma_{xx}$ results obtained for different material models for concrete. It should be noted that in the model with cracks analysis (marked as “cr”), the stress graphs are not smooth due to the occurrence of cracks in individual finite elements.

**Figure 13:**
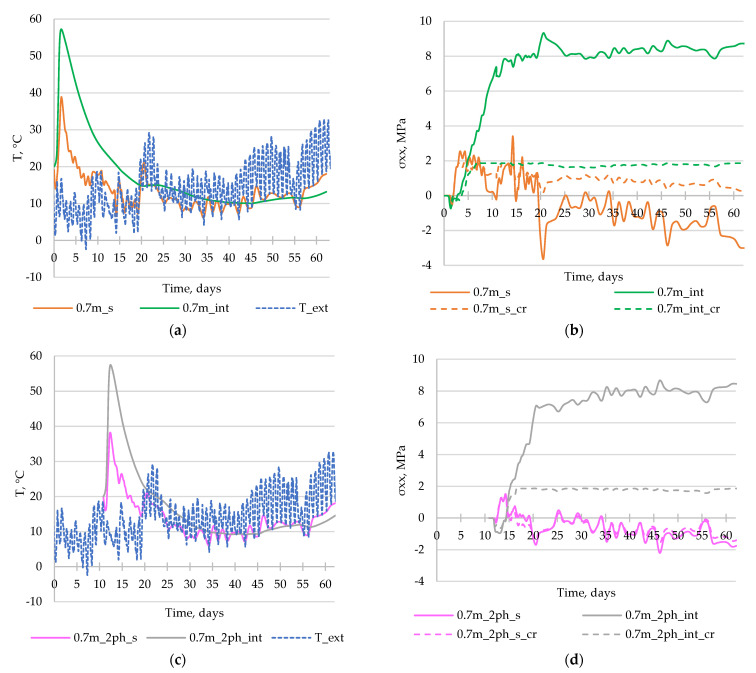
Development of (**a**,**c**) temperature and (**b**,**d**) stress $\sigma_{xx}$ in time at the crucial points (description in the text). T_ext–development of the external temperature (registered by the meteorological station in Opole; beginning of analysis: 22 March; interval: 1 h).

Figure 13 illustrates the development of temperature and stress during the hardening of the concrete wall. Points located approximately 0.7 m from the junction of the wall with the slab (stage 1) or with the previous stage (stage 2) were selected for this presentation. This point was chosen based on the stress distribution in the wall fixed to the foundation, which specificity results from both the uneven temperature distribution along the height of the wall and, more importantly, the change in the restraint coefficient along the wall height. Generally, this point is situated above the joint with the restraining element, although the exact location may vary in different cases [46]. As indicated in [32], the maximum stress location is closest to the construction joint for thin and short walls and increases with the wall’s thickness and length. In this study, for clarity, a critical point for analyzing temperature and stress development over time was selected at 20% of the height of each wall stage, corresponding to the maximum stress values obtained. This location also corroborated previous numerical analyses of walls with various thicknesses, lengths, and heights, showing that for similar length-to-height ratios, the maximum stress occurs at about 20% of the wall height [43]. Since maximum stresses occur at this level, graphs of temperature changes over time are also shown for the same points. Thus, Figure 13 presents the development of temperature and stress over time at the following points:

(a) Phase 1:
  - 0.7m_s: the point on the surface of the wall, located 0.7 m from the connection with the slab, model without cracking;
  - 0.7m_int: the point in the middle of the wall, located 0.7 m from the connection with the slab, model without cracking;
  - 0.7m_s_cr: the point on the surface of the wall, located 0.7 m from the connection with the slab, model with cracking;
  - 0.7m_int_cr: the point in the middle of the wall, located 0.7 m from the connection with the slab, model with cracking.
(b) Phase 2:
  - 0.7m_2ph_s: the point on the surface of the wall, located 0.7 m from the connection with the wall_1ph, model without cracking;
  - 0.7m_2ph_int: the point in the middle of the wall, located 0.7 m from the connection with the wall_1ph, model without cracking;
  - 0.7m_2ph_s_cr: the point on the surface of the wall, located 0.7 m from the connection with the wall_1ph, model with cracking;
  - 0.7m_2ph_int_cr: the point in the middle of the wall, located 0.7 m from the connection with the wall_1ph, model with cracking.

Figure 13a,c illustrates the temperature development in the model without cracking. Obviously, the same temperature development is in the model that took cracking into account. The presented diagrams provide additional information regarding the temperature changes, specifically highlighting the occurrence of a maximum temperature in the center of the wall (57.2 °C in the 1st phase and 57.4 °C in the 2nd phase of the wall) and the maximum temperature at the surface of the wall (37.35 °C in the 1st phase and 38.06 °C in the 2nd phase of the wall). It should be also noted that these values occurred earlier (1.5–2 days after casting) than the maximum temperature difference between the interior and surface was registered (3 days after casting). Furthermore, the presented temperature developments emphasize the noteworthy influence of the ambient temperature on the generated temperature. It is therefore imperative to accurately assign the ambient temperature when constructing the finite element (FE) model.

Figure 13b,d presents the stress evolution results, which are significantly dependent on the assumed material model. The difference is particularly noticeable in the case of stress occurring in the middle of the wall. Considering a model that incorporates cracking leads to stress development up to the point where the tensile strength of concrete is reached, resulting in a maximum stress value of less than 2 MPa. On the other hand, in the case of a material model without cracking, the stress development continues until it reaches a value of nearly 10 MPa.

**Figure 14:**
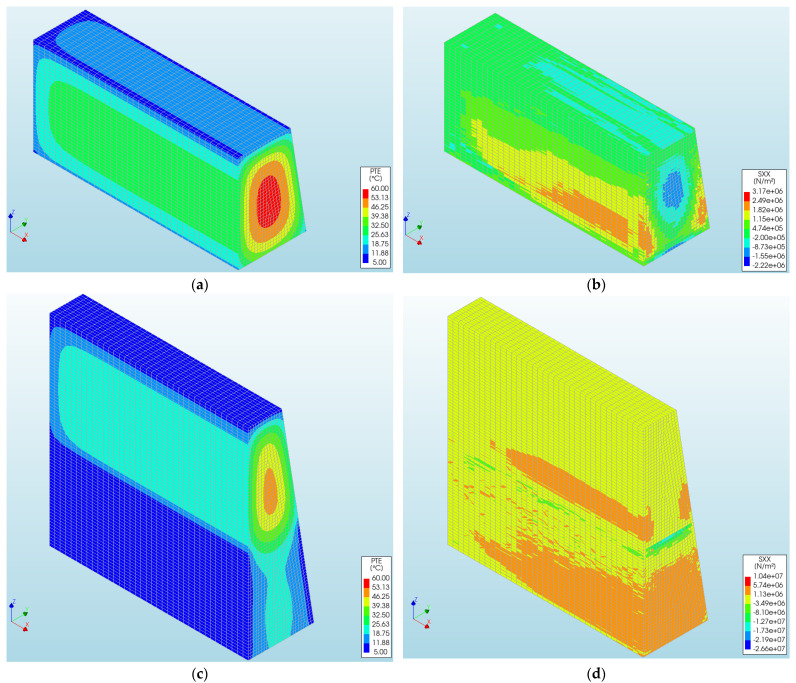
Maps of (**a**,**c**) temperature and (**b**,**d**) stress $\sigma_{xx}$ obtained in the 1st and 2nd phases of the wall for steps in which the maximum temperature was reached in particular parts of the wall.

FE modeling allows for the calculation of temperature and stress maps, offering a thorough understanding of stress magnitude and spatial patterns. Furthermore, these distributions are useful for identifying potential crack locations. Figure 14 showcases illustrative temperature and stress maps obtained during the 1st phase (Figure 14a,b) and 2nd phase (Figure 14c,d) of the wall, specifically at the moment when the maximum temperature is reached.

**Figure 15:**
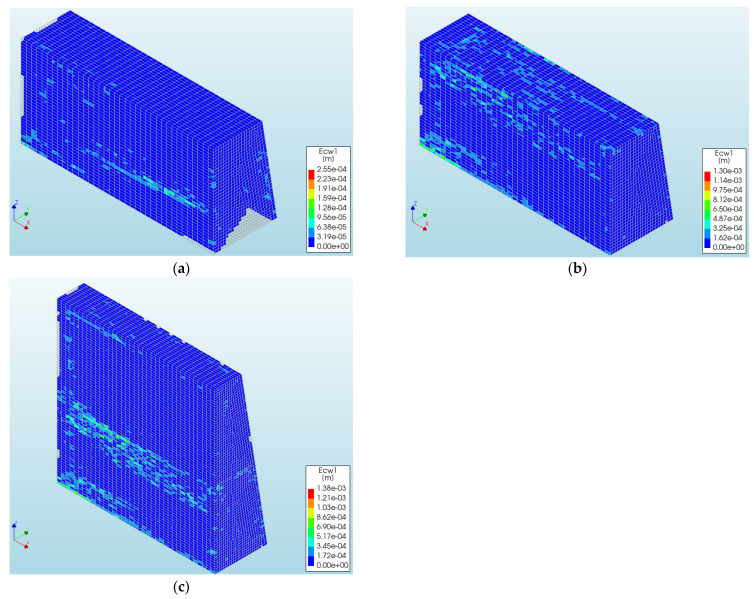
Maps of cracking obtained (**a**) 2 days after concreting the 1st phase, (**b**) 10 days after concreting the 1st phase (just before the 2nd phase), and (**c**) 30 days after concreting the 1st phase.

Using a cracked model for analysis also allows for the observation of the development of thermal cracks in the wall at individual calculation steps. While the stress development charts already partially show wall cracking, the maps offer a more comprehensive understanding of the cracking process. Illustrative maps from 2 days after concreting the 1st phase, 10 days after concreting (just before the 2nd phase), and 30 days after concreting the 1st phase are shown in Figure 15. In the following figures, cracks are formed in regions that are different from the dark blue color. After 2 days from casting, cracks form on the surface of the wall, about 0.7 m above the connection with the slab. These cracks correspond to the areas where maximum stress occurs. Later on, cracks appear on the higher parts of the lateral surface and on the top surface. Finally, as shown in Figure 15c, the widest cracks occur on the surface of the wall near the work break locations, such as the connection with the slab and between the 1st and 2nd phases of the wall. It is worth noting that the cracks visible on the maps are not typical for walls fixed in the foundation, where vertical cracks are usually observed. It can be explained by the fact that the observed crack pattern results from the combined effects of self-induced and restraint-induced strains and stresses. According to previous analyses, tensions of the self-induced origin in layers near the surface occur during the wall heating phase and turn into compressions during the cooling phase. External restraints, on the other hand, cause the entire wall to stretch during the cooling period. Only the detailed numerical analysis presented here, which considers the stages of concreting and the entire period from concreting to cooling of the wall, can capture these phenomena and provide a comprehensive view of the development of temperature, deformation, stress, and cracking. This example also clearly illustrates the complexity of performing such a finite element analysis, both in terms of inputting all the required data and evaluating the extensive results.

## 4. Conclusions

The proposed three-step method—comprising classification, simplified analysis, and detailed numerical modeling—provides a systematic approach to evaluate and mitigate early-age thermal effects in mass concrete structures. This structured methodology ensures that each step builds upon the previous one, leading to a comprehensive understanding and management of thermal cracking risks. The following steps are included in the proposed procedure:

- Step 1, with a preliminary assessment of the structure with the massivity index as well as an enhanced massivity index. The latter allows for a more accurate classification of structures based on their dimensions and expected thermal behavior, facilitating an initial assessment of whether a detailed analysis is necessary.
- Step 2, which involves straightforward analytical methods and enables the verification of initial predictions regarding self-heating temperatures. This step allows for the simple determination of temperature rise and strain during concrete hardening. If high temperatures and a risk of cracking are indicated, this justifies moving to the more detailed third step.
- For structures identified as having a high potential risk of early thermal cracks, detailed numerical modeling becomes essential in step 3. This step is particularly important for more complex structures, where accurate results require the use of advanced computational tools such as the Finite Element Method (FEM). These tools can simulate early-age concrete phenomena and assess the risk of cracking by identifying thermal–mechanical or thermal–moisture–mechanical effects. Various software can be used at this stage [38].

The exemplary analysis of a mass concrete wall constructed on a foundation illustrates the practical application of the proposed three-step method. This example underscores the method’s utility in real-world scenarios, providing a clear pathway from initial classification to detailed analysis and risk mitigation.

Moreover, the analysis reveals that despite using slag cement, significant temperatures in the wall and the risk of cracking may still occur. This is noteworthy because it challenges the common belief that substituting cement with slag cement alone is sufficient to mitigate the risk of thermal cracks. In the section related to step 3, the complexity of such an FE analysis is demonstrated, highlighting the importance of selecting appropriate computational data and parameters for predicting early-age thermal effects. It should be noted that in the absence of experimental results, certain literature data and analytical formulas can be utilized when inputting data [38]. The DIANA FEA 10.6 software, used as an example in this analysis, illustrates the methodology for conducting these detailed evaluations. It is important to note that the simplified analytical method evaluated maximum temperature values in the wall segments quite similar to those obtained through detailed numerical analysis. Indeed, for the first phase of wall concreting, the maximum center temperature was 59.17 °C determined by the analytical method and 57.2 °C determined using FEA, representing a 3.3% lower value. In the second wall stage, temperatures were 54.81 °C (analytically) and 57.4 °C (FEA), indicating a 5% higher FEA result. Surface temperature differences between analytical and numerical methods were more pronounced due to varying external temperatures modeled accurately in FEA versus a constant value in the simplified model. Surface temperatures were 33.13 °C (analytical) versus 37.35 °C (FEA) in stage 1 of the wall and 34.53 °C (analytical) versus 38.06 °C (FEA) in stage 2 of the wall, showing approximately 10–12% differences. Regarding strains and stresses, the analytical method assesses cracking risk without detailed crack opening analysis; thus, results in this area were not compared between the two methods. Nevertheless, the risk of cracking analytically predicted in step 2 was confirmed in step 3 by the numerical analysis, validating the effectiveness of the simplified analytical method.

Overall, this three-step method offers a comprehensive and practical framework for addressing early-age thermal effects in concrete structures, ensuring accurate assessments and effective mitigation strategies.

## Figures and Tables

**Table 1:** Proposed values of the coefficient $a_{Q}$ for chosen types of cement.

Type of Cement	$a_{Q}$
CEM I 42.5R	0.65
CEM II/B-V 32.5R	0.50
CEM II/B-S 32.5R	0.60
CEM III/A 42.5N-LH/HSR/NA	0.52
CEM V/A (S-V) 32.5R-LH	0.58
VLH V/B (S-V) 22.5	0.51

**Table 2:** Proposed values of total hydration heat $Q_{\infty }$ for chosen types of cement.

Type of Cement	Component, %			$Q_{\infty }$, kJ/kg
	Portland Clinker	Slag (S)	Siliceous Fly Ash (V)	
CEM I 42.5R	95.7	-	-	501
CEM II/B-V 32.5R	67.3	-	29.1	410
CEM II/B-S 32.5R	68.3	27.1	-	490
CEM III/A 42.5N-LH/HSR/NA	41.1	58.9	-	498
CEM V/A (S-V) 32.5R-LH	62.2	18.2	19.6	430
VLH V/B (S-V) 22.5	32.3	34.4	33.3	362

**Table 3:** Recommended values of coefficient χ for ordinary Portland cement CEM I.

Thickness of the Wall, m	CEM I
0.5	0.6
1.0	0.7
1.5	0.8
2.0	0.85
3.0	0.9

**Table 4:** Recommended values of coefficient χ for CEM II, CEM III, CEM V, and CEM VLH.

Thickness of the Wall, m	CEM II, CEM III, CEM V, and CEM VLH
0.5	0.45
1.0	0.57
1.5	0.7
2.0	0.8
3.0	0.85

**Table 5:** The coefficient of thermal expansion of concrete according to CIRIA C766 [5].

Aggregate Applied in Concrete *	$\alpha_{T}$ $,10^{-6}/{}^{\circ}C$
Basalt	10.5
Flint gravel	12
Quartzite	14
Granite	10.5
Limestone	9.5
Sandstone	12.5

* In the case of no information about the type of aggregate, the recommended value is $\alpha_{T}=12\cdot 10^{-6}/{}^{\circ}C$.

**Table 6:** Ultimate strains, $\varepsilon_{ctu}$, for concrete class C30/37 [5].

Coarse Aggregate Applied in Concrete *	$\varepsilon_{ctu}$ $\;\mathrm{after}\;3\;\mathrm{Days},\;10^{-6}$	$\varepsilon_{ctu}$ $\;\mathrm{after}\;28\;\mathrm{Days},\;10^{-6}$
Basalt	55	103
Flint gravel	60	112
Quartzite	66	123
Granite	66	123
Limestone	74	137
Sandstone	83	154

* In the case of no information about the applied type of aggregate, the recommended value of $\varepsilon_{ctu}$ should be assumed as for quartzite.

**Table 7:** The concrete mix of the structure.

Constituent	Amount, kg/m^3^
Sand 0/2	665
Basalt 2/8	595
Basalt 8/16	689
CEM III/A 42.5N-LH/HSR/NA	370
Water	152
Admixtures	6.14
Air	5%
w/c	0.42
Concrete class	C30/37
Density, kg/m^3^	2477

**Table 8:** Data for simple analytical calculations in step 2.

Description	Notation	Unit	Value	Comment
Thermal analysis				
Cement content	C	kg/m^3^	370	See Table 7
Coefficient	$a_{Q}$	-	0.52	See Table 1, Table 7
Total heat hydration heat	$Q_{\infty }$	kJ/kg	498	See Table 2, Table 7
Specific heat	$c_{b}$	kJ/(kg∙°C)	0.8	Based on data from [43]
Density of concrete	$\rho_{b}$	kg/m^3^	2477	See Table 7
Thickness: wall stage 1		m	2.17	See Figure 7; average thickness of the stage 1
Thickness: wall stage 2		m	1.57	See Figure 7; average thickness of the stage 2
Coefficient: wall stage 1	χ	-	0.81	See Table 4
Coefficient: wall stage 2	χ	-	0.72	See Table 4
Initial temperature	*T_bo_*	°C	20	Assumed ambient temperature +5 °C
Ambient temperature	*T_a_*	°C	15	Assumed spring conditions
Thermal transfer coefficient	α*_p_*	W/(m^2^ °C)	5.4	Before formwork removal
Thermal conductivity	$\lambda_{b}$	W/(m °C)	2.04	Based on data from Table 7 and [43]
Strain analysis				
Thermal deformability	α*_T_*	1/°C	10.5 × 10^−6^	Based on data from Table 7 and [5]
Stress relaxation	$K_{1}$	-	1	External restraint
Stress relaxation	$K_{1}$	-	0.65	Internal restraint (self-induced)
Restraint factor	R	-	0.5	External restraint, based on [49]
Restraint factor	R	-	0.42	Internal restraint, based on [5]

**Table 9:** Results from simple analytical calculations based on the procedure related to step 2.

Calculated Value	Wall_Stage 1	Wall_Stage 2	Comment
${∆T}^{adiab}$, °C	48.35		Equation (6)
${∆T}_{red}^{adiab}$, °C	39.17	34.81	Equation (7)
$T_{int}$, °C	59.17	54.81	Equation (8)
$T_{p}$, °C	33.13	34.53	Equation (10)
$T_{m}$, °C	50.49	50.06	Equation (11)
∆T, °C	35.49	33.05	Equation (12)
$∆T_{1}$, °C	26.04	20.29	Equation (13)
External restraint			
$\varepsilon_{r}$, με	186	174	Equation (14)
$\varepsilon_{ctu}$, με	55	55	Table 6
Cracking risk	Yes	Yes	Equation (16)
Internal restraint (self-induced)			
$\varepsilon_{r1}$, με	75	58	Equation (15)
$\varepsilon_{ctu}$, με	55	55	Table 6
Cracking risk	Yes	Yes	Equation (16)

**Table 10:** Basic material properties.

Property	Value		
	Concrete	Lean Concrete	Soil
Linear Material Properties			
E-modulus (28-day), N/m^2^	34.44 × 10^9^	15 × 10^9^	50 × 10^6^
Poisson’s ratio	0.2	0.2	0.2
Density, kg/m^3^	2477	2400	2070
Thermal expansion coefficient, 1/°C	1.05 × 10^−5^	1.2 × 10^−5^	1 × 10^−5^
Heat Flow			
Coefficient of thermal conductivity, W/(m∙°C)	2.04 (basalt)	1.7	1.4
Thermal capacity, J/(m^3^·°C)	1.892 × 10^6^ (basalt)	1.95 × 10^6^	2.15 × 10^6^

**Table 11:** Input data related to the material model considering cracking.

Property	Value
Tensile strength (28 days), MPa	1.86
Tensile curve	Linear-ultimate crack strain
Fracture energy, N/m	137.35

**Table 12:** Assumptions and input data for analysis related to the heat flow.

**Assumptions**		
Cement	CEM III/A 42,5N-HSR/NA	
Specific heat	0.8 (basalt)	kJ/(kgK)
Density	2477	kg/m^3^
Heat capacity c	1,892,000	J/(Km^3^)
Activation energy E_a_	38,500	J/mol
Gas constant R	8.314	J/mol/K
**Input data**		
Total produced heat per unit volume	8.1 × 10^7^	J/m^3^
Maximum value of heat production rate	5.05 × 10^9^	W/m^3^
Arrhenius constant	4630.7	K
Reference temperature	293.15	K

**Table 13:** Reinforcement provided in the analyzed structure.

Location	Amount of Reinforcement
top and bottom surface of the slab	Φ25 every 120 mm
lateral surfaces of the slab	horizontal bars: Φ25 every 100 mm
	vertical bars: Φ25 every 120 mm
lateral surfaces of the wall	Φ25 every 120 mm
top surface of the wall	Φ25 every 120 mm

**Table 14:** Parameters applied for Von Mises material model for reinforcement.

Material Parameter	Value
E-modulus, N/m^2^	211 × 10^9^
Thermal expansion coefficient, 1/°C	1.2 × 10^−5^
Yield stress, N/m^2^	5 × 10^8^

**Table 15:** Time of analysis.

Action	Duration
Casting of the slab	20 h
Slab analysis	28 days
Casting of the wall_ph1	18 h
Wall_ph1 analysis	10 days
Casting of the wall_ph2	10 h
Wall_ph2 analysis	51 days
**Total duration of the analysis**	**91 days**

**Table 16:** Heat transfer coefficient.

Connection	Heat Transfer Coefficient, W/(m^2^∙°C)
slab_lateral	5.4 * (first 14 days); 21 ** (after formwork removal)
slab_top	6 *** (first 28 days); 21 (after cover removal)
wallph1_lateral	5.4 (first 14 days); 21 (after formwork removal)
wallph1_top	21
wallph2_lateral	5.4 (first 14 days); 21 (after formwork removal)
wallph2_top	21
lean concrete	21
soil_top	21
remining surfaces	adiabatic conditions

* 5.4 W/(m^2^∙°C): for the formwork of 18 mm of plywood, with v = 5 m/s, ** 21 W/(m^2^∙°C): average value resulted from formulas provided in Table 17 (assuming v = 5 m/s), *** 6 W/(m^2^∙°C): cover of synthetic fabric.

**Table 17:** Review of literature proposals for prediction of the heat transfer coefficient, based on [48].

Source	Formula	Heat Transfer Coefficient
McAdams (1954) and Jonasson (1994)	$h_{T}=\left\{\begin{array}{l} 4.3\;V+6.2\;\;\;\;dla\;\;\;\;V\leq 5\;m/s\\ {7.6\;V}^{0.78}\;\;\;\;\;\;\;\;dla\;\;\;\;V>5\;m/s \end{array}\right\}$	27.7
Jayamaha et al. (quoted by Davies (2004))	$h_{T}=1.444\;V+4.955$	12.175
Silveira (1996)	$h_{T}=3.83\;V$	19.15
Branco et al. (1992)	$h_{T}=6.0+3.7\;V$	24.5

**Table 18:** Initial temperature assigned to the components of the model.

Element	Temperature, °C
slab and walls	20
lean concrete	10
soil: 0–2 m	8
soil: 2–10 m	6

**Table 19:** Mesh size.

Part of the Structure	Element Size
Slab	height: 0.1 m; plan view: 0.2 m
Wall	height: 0.1 m; thickness: 0.1 m; length: 0.2 m
Lean concrete	height: 0.075 m; plan view: 0.2 m
Soil 0–2 m	height: 0.5 m; plan view: 0.2 m (below the slab)–0.8 m (at the edges)
Soil 2–10 m	height: 0.8 m; plan view: 0.2 m (below the slab)–0.8 m (at the edges)

## Data Availability

The original contributions presented in the study are included in the article, further inquiries can be directed to the corresponding author.
